# A Reappraisal of the Threshold Hypothesis of Creativity and Intelligence

**DOI:** 10.3390/jintelligence8040038

**Published:** 2020-11-11

**Authors:** Selina Weiss, Diana Steger, Ulrich Schroeders, Oliver Wilhelm

**Affiliations:** 1 Institute of Psychology and Pedagogy, Ulm University, Albert-Einstein Allee 47, 89081 Ulm, Germany; diana.steger@uni-ulm.de (D.S.); oliver.wilhelm@uni-ulm.de (O.W.); 2 Institute of Psychology, University of Kassel, Holländische Strasse 36-38, 34127 Kassel, Germany; schroeders@psychologie.uni-kassel.de

**Keywords:** creativity, intelligence, threshold hypothesis, necessary but not sufficient condition

## Abstract

Intelligence has been declared as a necessary but not sufficient condition for creativity, which was subsequently (erroneously) translated into the so-called threshold hypothesis. This hypothesis predicts a change in the correlation between creativity and intelligence at around 1.33 standard deviations above the population mean. A closer inspection of previous inconclusive results suggests that the heterogeneity is mostly due to the use of suboptimal data analytical procedures. Herein, we applied and compared three methods that allowed us to handle intelligence as a continuous variable. In more detail, we examined the threshold of the creativity-intelligence relation with (a) scatterplots and heteroscedasticity analysis, (b) segmented regression analysis, and (c) local structural equation models in two multivariate studies (*N*_1_ = 456; *N*_2_ = 438). We found no evidence for the threshold hypothesis of creativity across different analytical procedures in both studies. Given the problematic history of the threshold hypothesis and its unequivocal rejection with appropriate multivariate methods, we recommend the total abandonment of the threshold.

## 1. Introduction

Can you be creative without being smart? Many researchers argued that creativity presupposes intelligence (e.g., Guilford 1967) and intuitively this proposition probably makes sense for many readers. Indeed, the abilities needed for divergent production/thinking (Guilford 1967) and idea generation and evaluation (Mumford and McIntosh 2017) are closely intertwined with other cognitive abilities, commonly referred to as convergent thinking (Carroll 1993; Cropley 2006). For example, the creativity required to come up with an invention for high-tech problems builds upon substantial expertise in a field, as well as decontextualized fluid intelligence (e.g., Nusbaum and Silvia 2011). However, the intellectual prerequisites for different tasks challenging creativity might vary (Diedrich et al. 2018; Jauk et al. 2013) and the relevance of general intelligence might not be the same at different points in the distribution of creative abilities.

Historically, creative ability was incorporated in most models of intelligence, predominantly as a lower-order factor below general intelligence. Creative abilities^1^—often measured by divergent thinking tasks, including indicators of fluency or originality (Runco 2008)—are part of the structure of intellect model (Guilford 1967), the three-stratum theory of cognitive abilities (Carroll 1993), and the Berlin intelligence structure model (Jäger et al. 1997; Süß and Beauducel 2005). The relation between intelligence and creativity was evaluated in several studies (e.g., in terms of a lower-order factor in the Cattell–Horn–Carroll model of cognitive abilities; McGrew 2009; Silvia et al. 2013). Recent evidence showed that creative abilities (e.g., divergent thinking scored for fluency) and general intelligence were substantially related (*r* = 0.46, Karwowski et al. 2018; *β* = 0.45, Nusbaum and Silvia 2011), especially when using a broad set of indicators (*β* = 0.51; Benedek et al. 2012; *β* = 0.40, Weiss et al. 2020a). This is corroborated by a review that states that the progress in analytical tools, as well as in measurement (e.g., in cognitive neuroscience), has led to the conclusion that creativity and intelligence are closely related (Silvia 2015). Research reporting lower correlations are often based either on narrow measures of the constructs or on very heterogenous measures (e.g., a meta-analysis by Kim (2005) found a mean correlation of *r* = 0.17). Among others, the substantial correlation between the two constructs resurrected the question if the relation between creativity and intelligence might not follow a necessary condition, but a necessary but not sufficient condition (Guilford 1967). Further, they wondered if it was in accordance with the so-called threshold hypothesis (e.g., Karwowski et al. 2016). In the present paper, we reviewed different interpretations of Guilford’s original finding and tried to translate them to testable statistical means. Moreover, we discussed three analytical approaches to study the relation between intelligence and creativity in two different data sets that varied with regard to the age of samples and the measures for creativity and intelligence. 

## 2. The Threshold Hypothesis of Creativity and Intelligence

**Figure 1:**
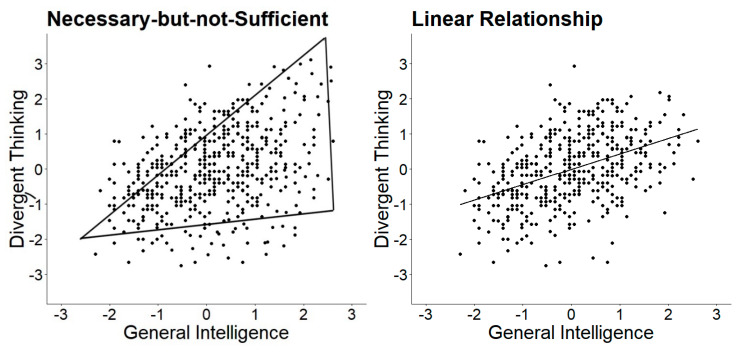

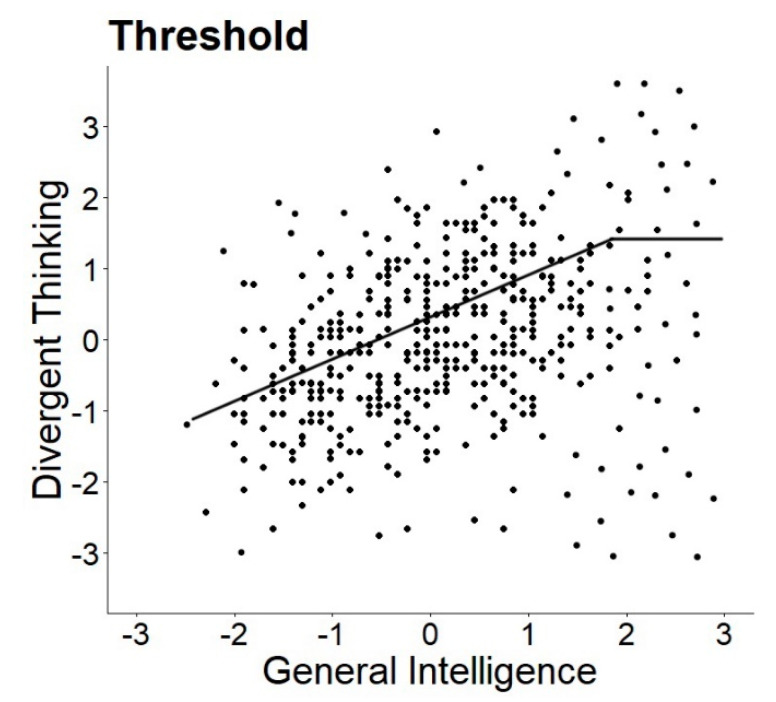
Schematic representations of the relation between creativity and intelligence. The *x*- and *y*-axis display *z* standardized values.

Guilford was one of the first to describe and investigate the relationship between creativity and intelligence. In his initial publication, he stated that “high IQ is not a sufficient condition for high DP [divergent production] ability; it is almost a necessary condition” (Guilford 1967, p. 168). Thus, Guilford assumed that highly intelligent individuals are not necessarily creative but can be creative, while less intelligent individuals are necessarily less creative (Guilford and Christensen 1973), which became an assumption known as the necessary but not sufficient condition. This relationship is schematically depicted in the left plot in Figure 1. If Guilford’s assumption holds and intelligence is a necessary but not sufficient condition for being creative, individuals’ scores scatter within the triangle. Although the original wording of Guilford’s theory was quite unambiguous, comparatively little research was done to test this assumption. Only recently, researchers picked up on the necessary but not sufficient condition (e.g., Karwowski et al. 2016; Shi et al. 2017). In contrast to the necessary but not sufficient condition, one can see that the necessary (and sufficient) condition corresponds to an ordinary linear regression (see Figure 1, middle plot).

The original formulation of a necessary but not sufficient condition was later (erroneously from many researchers) converted into the so-called threshold hypothesis. The threshold hypothesis states that the relationship between creativity and intelligence varies depending on the level of intelligence. Proponents assume that, below a certain threshold of intelligence, intelligence and creativity show a positive linear relationship, whereas above that threshold intelligence and creativity are uncorrelated (see right plot in Figure 1) or are less strongly correlated. Interestingly, although Guilford is widely named as the originator of the threshold hypothesis, he was no advocate in later publications and theoretically and analytically distinguished between the ideas of assuming a necessary but not sufficient condition, suggesting a threshold. Guilford and colleagues showed in two studies (including 45 tests of divergent production and two IQ tests with various scales) that none of the scatter plots suggested a threshold and that the ubiquitous positive relationship “shows a continuous, gradual shift from low to high IQ”, ultimately leading to the completely opposite conclusion that there is no support for any threshold (Guilford and Christensen 1973, p. 251). Guilford and Christensen concluded the absence of a threshold despite a triangular-shaped scatter for most of their plots (e.g., 20 triangular plots for semantic tasks out of 25 tasks), as the linear regression did not show any breaks. This implies that they distinguished intelligence as a necessary but not sufficient condition for being creative (triangular shape of a scatterplot) and the assumption of a threshold given by a difference in correlations between creativity and intelligence tasks at a certain point (break in the regression line; see Figure 1). In summary, there are (at least) three different perspectives on the link between creativity and intelligence: intelligence being (a) a necessary condition, (b) a necessary but not sufficient condition, and (c) the threshold hypothesis. Herein, we overview what researchers understand by the term “partly vary”. In the following, we discuss the theoretical assumptions and empirical evidence of the threshold hypothesis.

## 3. Theoretical Underpinnings of the Threshold Hypothesis

What are the theoretical underpinnings for the threshold hypothesis? Unfortunately, a large amount of research regarding the intelligence-creativity link lacks a thorough theoretical explanation as to why a threshold should exist and if present where it should be (see Karwowski and Gralewski 2013). The confusion of terms and the different operationalizations to test the theory might be a direct result of sparse theoretical ideas. However, to discuss where a threshold should exactly be placed in the ability distribution is irrelevant if the “why” is not clear. Although the threshold hypothesis could not be equated with a non-linear relationship between intelligence and creativity, some researchers borrow the theoretical argumentation from other parts of intelligence research, i.e., the ability of the dedifferentiation hypothesis (i.e., Spearman’s law of diminishing returns (SLODR, Spearman 1927) or age-related differentiation (Garrett 1946)) to explain the threshold hypothesis of creativity.

At first glance, lending ideas from SLODR seem to be a viable approach, as (general) intelligence directly affects the ability to be creative (e.g., Forthmann et al. 2019; Gilhooly et al. 2007; Silvia et al. 2013). According to SLODR (Spearman 1927), correlations between cognitive abilities decrease with increasing levels of abilities (e.g., Hartung et al. 2018). Transferring this logic would imply that intelligence might facilitate the use of elemental skills (e.g., long-term memory) and, once an advanced level of intelligence is reached, higher levels of intelligence are no longer beneficial for further increasing creative performance, thus leading to a correlational pattern as discussed above. A further example can be found in the differentiation of language ability (Garrett 1946). Initially, it depends on single skills such as oral language comprehension, but the more mature someone gets the more language abilities are differentiated (e.g., reading comprehension, linguistic usage). However, the evidence regarding age differentiation is mixed (Breit et al. 2020; Van Der Maas et al. 2006), and theoretical explanations for this phenomenon are surprisingly sparsely elaborated upon. Some findings support ability differentiation (e.g., Legree et al. 1996), while others use more sophisticated data-analytic approaches to see support for the differentiation hypothesis (Hartung et al. 2018).

However, the consideration of this literature only adds little insight when it comes to why there should be a qualitative gap or threshold in the relation of creativity and intelligence. Moreover, the literature does not provide any cohesive theoretical background for where to set a cutoff a priori. Despite this weak theoretical foundation of the threshold hypothesis, the question if there is a threshold still inspired a considerable amount of studies. In the next paragraph, we summarize the empirical evidence from these studies and give a systematic overview of the findings.

## 4. Empirical Evaluation of the Threshold Hypothesis

In Table 1, we summarize prominent findings on the threshold hypothesis and give an overview the methods and results of the studies. Strikingly, almost as many different thresholds existed as did studies. The diverse set of results can be attributed to (a) different understandings how the threshold hypothesis is best operationalized, (b) varying sample sizes and sample characteristics, (c) different measures used to assess both intelligence and creative ability, and (d) the analytical procedures to settle a specific threshold. 

Although the sample size reported in the studies varied considerably (e.g., *N* = 88 to *N* = 12,255), sample size did not seem to affect the results systematically, leaving no evidence for potential publication biases due to missing statistical power. The same was true for other sample features, although some may argue that sample characteristics such as age or ability distribution might influence the results. Age itself had been assumed to affect the factor structure of intelligence as stated in the age differentiation hypothesis (Garrett 1946), but findings were mixed (Breit et al. 2020; Hülür et al. 2011; Tucker-Drob 2009). Moreover, as the threshold was assumed to be at an intelligence score around *z* = 1.33, some samples might have simply failed to include enough cases above that threshold, failing to depict the whole ability spectrum. However, on the contrary, the studies reported in Table 1 show the opposite effect. Studies that oversampled highly gifted participants (*z* > 2, Holling and Kuhn 2008; *z* > 1.33, Preckel et al. 2006) did not find evidence for the threshold hypothesis.

Second, the measures used to study the threshold hypothesis might have influenced the results. For example, Jauk et al. (2013) derived varying thresholds for different dimensions of divergent thinking (originality: *z* = 0, creative fluency: *z* = 1.33; ideational fluency: *z* = −1.00), but no threshold for the relation between creative achievement (assessed via self-reports) and intelligence. Overall, the measures of creativity used in the different studies differed largely in breadth and depth of their operationalization (Weiss et al. 2020b). With respect to the measures of intelligence, most studies focused on indicators that assessed fluid intelligence—the ability of abstract reasoning in novel situations—which is an important constituent of overall general intelligence (e.g., Heitz et al. 2005). It is recommended to use a broad measure of creativity when assessing the threshold to eliminate potential item selection bias from narrow tests, although no systematic influence was established (Table 1).

In contrast to the aforementioned study characteristics, the analytical strategy affects whether and where a threshold is found (e.g., Karwowski and Gralewski 2013). Both correlational analyses and segmented regression analyses mostly reported the existence of a threshold (e.g., Cho et al. 2010; Jauk et al. 2013), which varied. Two studies that used correlational analyses confirmed a threshold at *z* = 1.33, despite segmented regression analysis often resulting in different thresholds. Conversely, multi-group confirmatory factor analysis, which evaluates the factor structure (of creativity) in different ability groups, seemed to show no difference between the groups (Holling and Kuhn 2008; Preckel et al. 2006). Based on the previous results, it seemed plausible that the analytical method had a direct impact on the results. Therefore, we considered different methods to probe the threshold hypothesis. 

## 5. Analytical Strategies in the Investigation of the Threshold Hypothesis

Previous studies reported results regarding the threshold hypothesis, most of which were based on a (a) correlational analysis in a split sample, (b) segmented regression analysis, and (c) multi-group confirmatory factor analysis. Additionally, the necessary but not sufficient condition analysis (for more information, see Dul 2016) has recently gained attention as a statistical tool in the threshold literature. However, the results of the necessary but not sufficient condition analysis could not be directly compared to the results of other methods. Finding a significant proportion above the ceiling did not necessarily imply a threshold (Guilford and Christensen 1973; Ilagan and Patungan 2018), because it did not test for a break in the regression line (see Figure 1). Moreover, there were several open theoretical issues (e.g., causality assumptions that are not examined and further problematized) and issues regarding that method (e.g., no account for sampling error and a high sensitivity to outliers; for a criticism see Ilagan and Patungan 2018). In the present paper, we focused on methods that were used to study the threshold hypothesis rather than the necessary but not sufficient condition. 

### 5.1. Correlational Analysis in Split Sample

The correlational analysis—which often capitalizes on an extreme group design (Preacher et al. 2005)—is the analytical method with the longest tradition in the investigation of the threshold hypothesis (e.g., Cho et al. 2010; Fuchs-Beauchamp et al. 1993; Getzels and Jackson 1962). For this analytical approach, the sample is split into two groups at an a priori set threshold into a low ability group and a high ability group with correlations between intelligence and creativity separately computed. According to the threshold hypothesis, a threshold exists if the correlation is lower or even zero in the high ability group compare to the low ability group (Karwowski and Gralewski 2013). Although this method might seem like a direct translation of the threshold hypothesis into statistical means, it comes with a long list of potential disadvantages. First, the sample split needs a strong theoretical justification for setting the threshold. Given the unclear theoretical roots of the threshold hypothesis, the often-used threshold of *z* = 1.33 is not sufficiently backed up by theory. Eventually, this uncertainty concerning the cutoff yields the risk of exploiting researcher’s degrees of freedom (Simmons et al. 2011; Wicherts et al. 2016), probing different thresholds until the desired result is achieved. Second, splitting the sample into two subsamples dichotomizes an otherwise continuous variable (i.e., intelligence), which results in all sorts of statistical problems, such as informational loss, an underestimation of the strength of the bivariate relation, and a mis-categorization of participants that are close to the threshold (MacCallum et al. 2002). Third, as the correlational analysis is based on manifest variables, measurement error and task specificity are not taken into account. Fourth, the analysis most likely suffers from a lack of measurement precision at the more extreme points of the ability distribution because fewer items assess the extremes (Byrne 2010). Fifth, such differences in the correlational patterns in two groups are often biased by samples restricted in dispersion and reliability being lower in the group that is more severely range-restricted. Since the high IQ group in a heterogeneous sample for obvious reasons often contains only a few cases, the parameter estimates (e.g., slope of the regression) are less robust. The point estimate is lower by virtue of variance restriction and by virtue of the fact that item difficulty distribution often follows ability distribution. Therefore, fewer items with good discrimination are available in the tails of the distribution. This indicates that the reliability of person parameters follows the test information function, which is low where few items discriminate. Therefore, sufficient statistical power can often not be reached in extreme groups of small sizes. Consequently, correlational analysis is especially prone to false positive conclusions due to the very nature of the threshold hypothesis.

### 5.2. Segmented Regression Analysis

Segmented linear regression analysis determines whether different (linear) relationships exist across the continuum of intelligence. This regression analysis includes the estimation of multiple linear models that are fitted for different segments of the data (Ryan and Porth 2007). This means the intelligence continuum is divided several times into two segments and ordinary least squares (OLS) regressions are fitted separately within these segments. A break, which is referred to as a threshold in the linear regression (such as displayed in Figure 1, right panel), means that the slopes of the two regressions differ significantly. A possible advantage of this method is that it can be used to detect a potentially unknown breakpoint rather than confirming an a priori set breakpoint (Ryan and Porth 2007). The method is usually applied if there is a strong theoretical assumption that justifies a break in the relation often in terms of a dose-response relationship (e.g., a critical level of stress leads to preterm birth, Whitehead et al. 2002). Such a strong theoretical basis cannot be assumed in the relation between intelligence and creativity. Furthermore, the segmented regression comes along with several model assumptions that normally distributed and independent residuals are homoscedastic. i.e., OLS regression (Ryan and Porth 2007). However, studies reporting results based on the segmented regression analysis often fail to report tests of homoscedasticity of the data or QQ-plots that examine the normal distribution of residuals. We chose a segmented regression analysis to allow a direct comparison to previous research and because the basic assumptions were met (i.e., homoscedasticity of residuals). Robust alternatives to segmented regression, such as the robust bent line regression (Zhang and Li 2017) or the Robin Hood algorithm for curvilinear relations (Simonsohn 2018), can be considered if the assumptions are violated. These analytical methods assume an unknown change point in a non-linear regression of manifest variables, but the theoretical basis for such an assumption is vague. Moreover, these methods also suffer from problems such as imprecise false positive rates (Type I errors) and the assumption of a change in sign of the regression in two regions (Simonsohn 2018). Besides, methods such as the quantile regression have been applied to investigate thresholds (Dumas 2018; Karwowski et al. 2020), although they do not provide a direct test of a threshold as the segmented regression analysis does.

### 5.3. Local Structural Equation Modeling

The last analytical method we want to present is a novel approach, termed local structural equation models (LSEM; Hildebrandt et al. 2016). To understand its merits, we will first address the shortcomings of multi-group confirmatory factor analysis (MGCFA), which has been previously used in the threshold literature. MGCFA is a method within the framework of structural equation modeling to analyze measurement parameters (e.g., factor loadings, item intercepts) across different ability groups beyond a simple comparison of correlations (Vandenberg and Lance 2000). Although the latent variable approach is superior compared to simple regressions of manifest variables in an extreme group design, the multi-group setting requires an arbitrary dichotomization of a continuous variable (e.g., z = 2.00, Holling and Kuhn 2008; z = 1.33, Preckel et al. 2006). Another disadvantage of the method is that it does not allow for the direct examination of the correlation of creativity and intelligence, as well as its change across the intelligence continuum. In general, studying the factor variance of creativity over the intelligence continuum might indicate a notable change or threshold (e.g., Holling and Kuhn 2008), i.e., a systematic increase or decrease in factor variance is one way that (de-)differentiation can manifest (Molenaar et al. 2010). However, multi-group confirmatory factor analyses that rely on discretizing a continuous variable at an arbitrary point can mask such a change in the variance. A recent extension of the structural equation models that ameliorates the drawback of an artificial dichotomization of the continuous variable intelligence is LSEM (Hildebrandt et al. 2016). In a nutshell, LSEM involves the fitting of several “conventional” structural equation models along the distribution of a continuous moderator with weighted observations (Olaru et al. 2019). The weight of each observation is based on the proximity of an observation to a specific value of the moderator, so that observations near this focal point provide more information to model estimation than more distant points. In the present context, a series of measurement models for creativity was estimated with intelligence as a continuous moderator. Based on this method, changes in the model fit the factor structure, mean values, and variances without splitting the sample into arbitrary groups (see for example Hartung et al. 2020). 

## 6. The Present Studies

The threshold hypothesis is often attributed to Guilford, though he intended for a necessary but not sufficient condition between intelligence and creativity. In fact, he opposed the idea of a threshold based on empirical findings (1973). Since then, the threshold hypothesis has developed a life of its own, despite the empirical support being weak. In our reading, the theoretical basis of the cognitive mechanisms of the threshold hypothesis, as well as the data analytical approaches, are often not met with the necessary rigor. Applying Occam’s razor, no threshold should be assumed or postulated unless convincingly demonstrated otherwise. In the present manuscript, we re-analyzed two data sets that varied with respect to participants’ age and the indicators of creativity and intelligence with different analytical strategies. More specifically, we evaluated the relation between intelligence and creativity in both data sets based on the following analytical strategies: (a) scatterplots and heteroscedasticity analysis, (b) segmented regression analysis, and (c) local structural equation models. 

## 7. Method

### 7.1. Samples and Design

#### 7.1.1. Study 1

The first data set included measures of intelligence, emotional intelligence, and creativity. It was published in the context of investigating the self-other knowledge asymmetry (Neubauer et al. 2018). After data cleaning (excluding *n* = 6 multivariate outliers with a Mahalanobis distance > 15; Meade and Craig 2012), the total sample included *N* = 456 adolescents and young adults (ranging from 13 years to 20 years). About 55% of the participants were female. The students were recruited from 13 different public and private schools in rural and urban areas of Austria. For more information please see Neubauer et al. (2018). The dataset is available online via OSF (https://osf.io/v8e5x/).

#### 7.1.2. Study 2

The second data set was part of a larger multivariate study of creativity and its covariates (Goecke et al. 2020; Steger et al. 2020; Weiss et al. 2020a). The analysis was based on *N* = 438 participants after excluding *n* = 12 multivariate outliers with a Mahalanobis distance > 15. Two participants showed high-end performance regarding all creativity indicators. They were not excluded from the data set as they were not flagged as multivariate outliers. The sample included adults between 18 and 49 years. About 65% of the participants were female. For more information regarding the sample and data preparation, see Weiss et al. (2020a). The dataset is available online via OSF (https://osf.io/6fxv5/).

### 7.2. Measures and Scoring 

#### 7.2.1. Study 1 

In the first study by Neubauer et al. (2018), intelligence was measured based on the “Intelligenz-Struktur-Analyse” (ISA; Fay et al. 2001), which includes three subtests for verbal, numerical, and spatial reasoning. Creativity was measured using three items from the “Alternate Uses Task” (Jauk et al. 2013). Participants were instructed to name as many original alternate uses for an umbrella, plastic bottle, and a shoe as possible within two minutes. We presented the results for the fluency scoring of answers, i.e., the human coding of the quantity of solutions (for more information, see Neubauer et al. 2018). The fluency scores matched the instruction, which were highly correlated with originality score and frequently applied in the literature. Additionally, we also present the results based on originality scores in the supplementary material (Figures S2–S4). 

#### 7.2.2. Study 2

In the second study, intelligence was measured using the verbal and figural subtest of the “Berlin Test of Fluid and Crystallized Intelligence” (Wilhelm et al. 2014). Divergent thinking was measured based on six verbal and figural tests that were either instructed for fluency or originality. The similar attributes test (including 6 items) and the inventing names test (including 18 items) were both adapted from verbal creativity tests (Schoppe 1975). The other two fluency indicators were a typical retrieval fluency test (including 6 items), and the figural fluency test (including 4 items; Jäger et al. 1997). All fluency indicators were rated by humans for the frequency of solutions. Two additional tests (combining objects, French et al. 1963) and inventing nicknames (Schoppe 1975) were rated for the originality/creativity of solutions. Three human raters scored participants’ answers on a five-point rating scale (Amabile 1982; Silvia et al. 2008). For more detailed information, please see Weiss et al. (2020a).

### 7.3. Statistical Analyses

The heteroscedasticity analysis and segmented regression analysis were based on manifest variables. We used *z*-standardized mean values, including either all creativity indicators (Study 1: three items of the Alternate Use Task; Study 2: six tests of fluency and originality) or all intelligence indicators (Study 1: three subtests for verbal, figural, and numerical fluid intelligence; Study 2: indicators for figural and verbal fluid intelligence). The local structural equation modeling relies on a measurement model for creativity using *z*-standardized values. In Study 1, the measurement model was identified using three indicators of the alternate uses task, whereas the model fitted the data well in Study 2 (χ^2^(9) = 13.31, *p* = 0.15, CFI = 0.99, RMSEA = 0.03, SRMR = 0.03). In comparison to Weiss et al. (2020a), we modeled creativity as a single factor of divergent thinking, excluding the nested originality factor in the present analysis because it shows low factor saturation and factor variance, which causes estimation problems in LSEM. 

#### 7.3.1. Scatterplots and Heteroscedasticity 

First, we investigated whether a threshold existed using a scatterplot analysis. Since visual inspection of scatterplots is highly subjective, we tested for heteroscedasticity. Normally distributed residuals indicate homoscedasticity, i.e., the absence of heteroscedasticity. We assumed that if a somehow non-linear relationship between creativity and intelligence existed, values should show a heteroscedasticity, which could be tested with the Breusch–Pagan test (Breusch and Pagan 1979). The Breusch–Pagan test assumes a constant confounding variable variance in the null hypothesis. A non-significant test for heteroscedasticity rendered the existence of a threshold very unlikely.

#### 7.3.2. Segmented Regression Analysis 

We used the segmented regression analysis as a second approach to investigate the threshold hypothesis. In this case, a significant change in the slope of the linear regression within the two segments indicated the existence of a threshold. In both studies, intelligence was analyzed as independent variable and divergent thinking as the dependent variable. In addition to estimating the amount and position of possible breakpoints, we used the Davies test to see if any breakpoints occurred between the second greatest and second smallest value (Davies 2002; Muggeo 2008). As no significant changes were assumed if more than 10 segments were specified, we used the recommended default of the Davies test (i.e., 10 segments). If the Davies test was non-significant, the regression parameters were constant across the complete intelligence range. 

#### 7.3.3. Local Structural Equation Modeling

**Figure 2:**
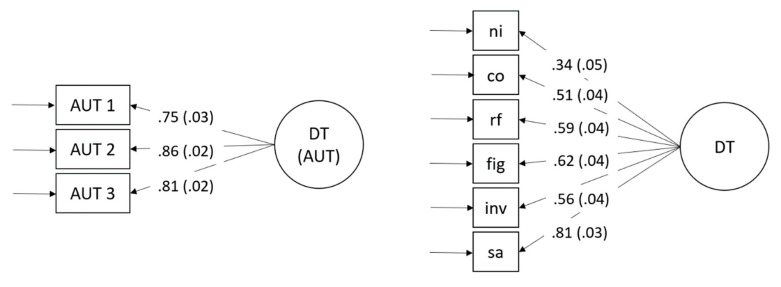
Measurement models for divergent thinking. Study 1 (**left model**), Study 2 (**right model**) including standardized loadings and standard errors. Study 1: AUT are single items of the alternate uses task. Study 2: indicators are test-scores. Fluency test-scores are as follows: sa (similar attributes), in (inventing names), ff (figural fluency), and rf (retrieval fluency). Co (combining objects) and ni (nicknames) are originality indicators that were only instructed and scored for originality.

Finally, we used LSEM to investigate the threshold hypothesis. In contrast to MGCFA, which relies on the categorization of intelligence as a moderator (e.g., Holling and Kuhn 2008), LSEM allows for the investigation of a factor structure of creativity (Figure 2) across the intelligence continuum. LSEM is a person-sampling method applied to investigate deviations in the measurement model across observations (Olaru et al. 2019). Compared to MGCFA, which requires the grouping of participants, the observations in LSEM are weighted as a function of their proximity to a focal point of intelligence (Hildebrandt et al. 2009). The weights are normally distributed around the focal point, implying a full weight at a focal point and weights decreasing according to the probability density of the normal distribution with increasing distance from the focal point. For example, if the measurement model of divergent thinking (Figure 2) is estimated at the focal point of *z* = 1.33, all participants with an intelligence score of *z* = 1.33 are assigned the highest weight (i.e., 1), and weights decrease as scores are more distant from *z* = 1.33. For each focal point of intelligence, the measurement model of creativity is sequentially estimated based on the weighted samples (Hildebrandt et al. 2016). In Studies 1 and 2, we applied general intelligence as a moderator based on a moderator grid of *z* = 0.5, ranging from *z* = −1.50 to *z* = 1.50, resulting in seven focal points. The effective sample size ranged between *N_eff_* ≈ 106 and *N_eff_* ≈ 215 for Study 1 and *N_eff_* ≈ 92 and *N_eff_* ≈ 223 for Study 2. 

### 7.4. Open Science

We conducted all analyses using R version 4.0.2. Segmented regression analyses were estimated using the R package segmented (Muggeo 2008), whereas LSEM was conducted using the packages lavaan and sirt (Robitzsch 2020; Rosseel 2012). To make the present analyses transparent and reproducible, we provided all material (i.e., data set of Study 2, syntax, and supplemental material) at the Open Science Framework. The data set of Study 1 is available online. We also report descriptive statistics (i.e., mean values, standard deviations, and correlations) for the indicators used in the following analysis in the supplementary material (Table S1 and Figure S1). 

## 8. Results 

### 8.1. Scatterplots and Heteroscedasticity

**Figure 3:**
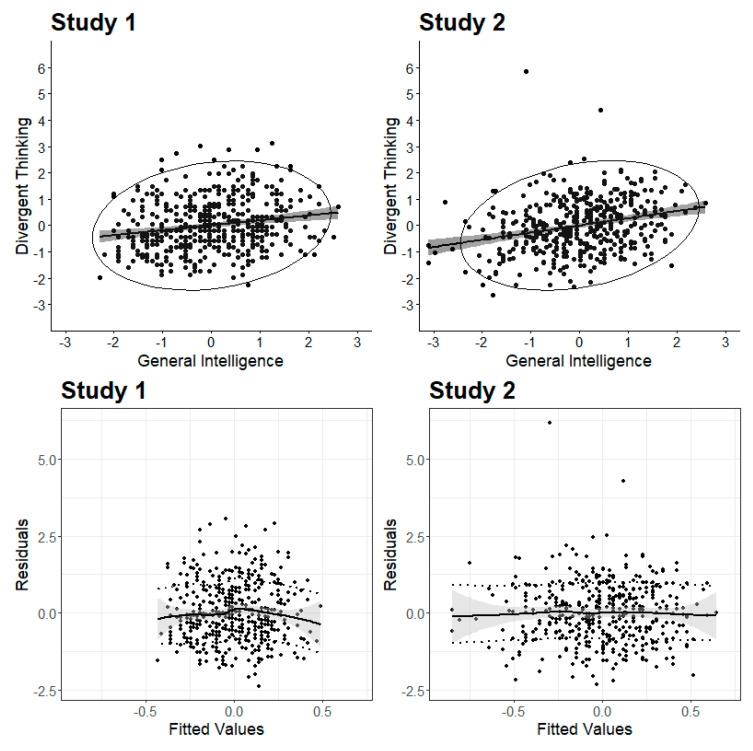
Scatterplots and heteroscedasticity plots. Scatterplots (including the 95% confidence interval) for the correlation between divergent thinking and intelligence are presented upper part. Heteroscedasticity plots including standard errors (grey) and standard deviations of the fitted values (dashed line) are given in the lower part.

Scatterplots and testing for heteroscedasticity were ur first means to investigate the datasets and to skim for breakpoints in the relation between creativity and intelligence (see Figure 3). In Study 1, the correlation between creativity and intelligence was lower (*r* = 0.19, *p* < 0.01) than in Study 2 (*r* = 0.27, *p* < 0.01). At first glance, the scatterplots (Figure 3, upper part) showed no sign of a threshold. The heteroscedasticity plots (Figure 3, lower part) showed flat lines based on the loess smoothing function, which indicated evenly distributed residuals across the fitted values. Additionally, the Breusch–Pagan test for heteroscedasticity was not significant in both studies (Study 1: BP(1) = 0.64, *p* = 0.42; Study 2: BP(1) = 1.16, *p* = 0.28), so that homoscedasticity could be assumed. The scatterplot and heteroscedasticity plot based on the originality scores (Study 1) are presented in the supplementary material (Figure S2). The Breusch–Pagan test was not significant for originality (BP(1) = 0.47, *p* = 0.49).

### 8.2. Segmented Regression Analysis 

**Figure 4:**
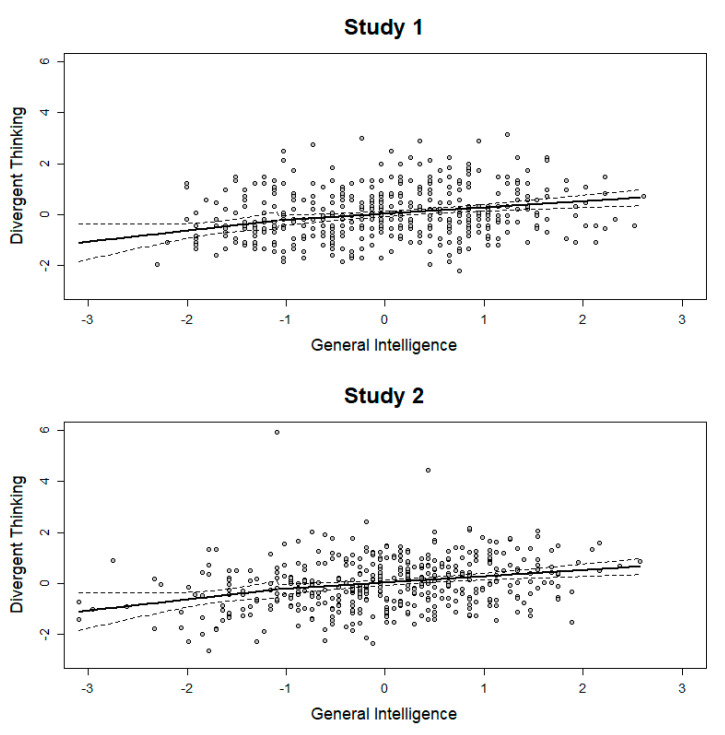
Segmented regression analysis. The breakpoint for the relation between general intelligence and divergent thinking. The dotted line represents the 95% confidence interval.

The segmented regression analysis estimates breakpoints in an otherwise linear relationship between two variables. For all breakpoints, the change in slope were not significant; Figure 4 displays the largest change in slopes for Studies 1 and 2. The largest change in slope for the originality indicators (Study 1) is presented in the supplementary material (Figure S3), which was not significant. In sum, there is no evidence for the threshold hypothesis using segmented regression analysis. Nevertheless, we estimated Δ*R*^2^ on Fisher’s *z*-standardized correlation coefficients with *z* = 1.33 as a breakpoint, because this cutoff was often selected as a potential threshold. In both studies, the number of participants after the breakpoint was small (*n*_1_ = 47, *n*_2_ = 43). The resulting difference was Δ*R*^2^ = 0.06 in Study 1 and Δ*R*^2^ = 0.05 in Study 2. 

### 8.3. Local Structural Equation Models 

**Figure 5:**
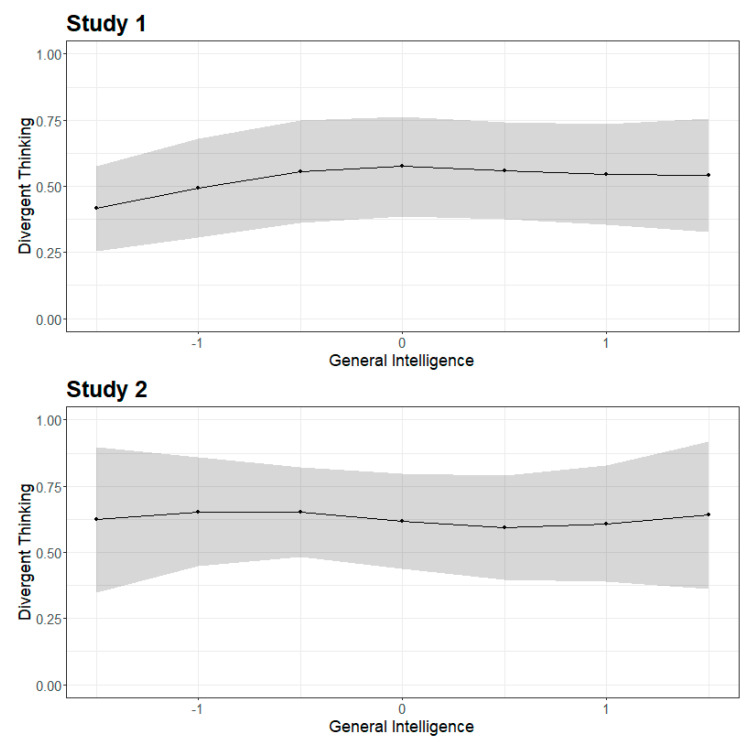
Standardized factor variances at each focal point along the intelligence continuum.

To detect possible changes in the factor variance of creativity along the intelligence continuum as an indication of a threshold, we fitted local structural equation models in Studies 1 and 2. The model in Study 1 was identified, while the measurement model for creativity fitted well along the intelligence continuum in Study 2, with a slight deterioration in model fit at the tales of the distribution (CFI_min_ = 0.92, RMSEA_max_ = 0.10, and SRMR_max_ = 0.05). No systematic changes in the factor variance of divergent thinking across general intelligence as a moderator were detectable (see Figure 5; see Figure S4 in the supplement for changes in the factor variance of originality). Furthermore, we also fitted a model that constrained the factor loadings to equality to examine if model fit deteriorates. The constraints were introduced to the model with the joint estimation approach for LSEM (separate models at the focal points are equivalently estimated in a multiple group model context; implemented in sirt::lsem.estimate). Similar factor loadings and no decrement in the model fit contradict the idea of a threshold. The loadings at the different focal points in Studies 1 and 2 are displayed in the supplementary material (Study 1: Figure S5; Study 2: Figure S6). As there was no significant change in the model fit, it can be assumed that the loadings do not show greater changes at different focal points in both studies.

## 9. Discussion

Investigations regarding a change in the relation between variables above and below a threshold are not limited to creativity research, but can be encountered in many fields such as second language learning (e.g., Cummins 1979). In our reading, these threshold-hypotheses share that they are overgeneralizations of evidence that mainly derived from studies with small sample sizes. Besides, these studies often lacked comprehensive theoretical underpinnings, which is in stark contrast to the extensive attention these hypotheses have attracted over the past decades. Thresholds assumptions should be encountered with some skepticism steered by various conceptual and methodological problems (Takakuwa 2003), and should entail some essential questions. 

### 9.1. Does a Threshold Exist? 

In the present case, we reanalyzed two studies with different operationalizations of both fluency/originality and intelligence using three analytical approaches to investigate a potential threshold. Despite these efforts, we were unable to find any compelling evidence for the existence of a threshold. First, the scatterplots of intelligence and creativity did not show any abnormalities and the data were homoscedastic. Second, we found no significant breakpoints using the segmented regression analysis. Finally, the factor variance and factor loadings of a measurement model of creativity did not change across the intelligence continuum. Moreover, since our findings were based on relatively large sample sizes, including different age groups and a variety of different measures of both constructs, we deemed it unlikely that our results were distorted due to a lack of power or sampling issues. This finding is congruent with a number of previous studies that were also unable to find support for an intelligence creativity threshold (e.g., Preckel et al. 2006; Sligh et al. 2005). Remember that Guilford himself led the way when he concluded from two large multivariate studies that he found “no evidence to support a threshold hypothesis regarding the relation of creative potential to IQ” (Guilford and Christensen 1973, p. 252). We concur with this statement. Despite our systematic approach, we did not find any evidence to support the existence of a threshold.

### 9.2. Why Do Researchers Keep on Finding Evidence Anyway? 

While the inference drawn from our results is unambiguous, previous research on the existence of a threshold of creativity and intelligence is not. A narrative review might infer that the results are mixed with some evidence against a threshold (e.g., Holling and Kuhn 2008; Preckel et al. 2006), and some evidence in favor of a threshold (e.g., Jauk et al. 2013; Karwowski et al. 2016). What are potential causes for these inconclusive results? As we saw in the literature review, some differences were caused by the choice of specific analytical approaches, yet the problem goes deeper. Maybe the most apparent is that the threshold is not set a priori. Short of a convincing theory, these cutoffs are arbitrary and leave ample room for many researchers’ degrees of freedom in the data analysis. This problem is exacerbated by different handlings of outliers, the choice of analytical tools, etc. (Simmons et al. 2011). Declaring a threshold presupposes its existence and a specific number suggests a precision rarely found in behavioral sciences. As such, it neglects its positivistic identification. Thresholds suggest a qualitative difference of humans below and above the value that is implausible with respect to creativity and intelligence in specific but also, more generally, for psychological dispositions. Even more nuanced approaches—such as the conditional threshold theory (Harris et al. 2019), that supposes that openness plays a critical role in the intelligence-creativity threshold—are adding further complexity and researcher’s degrees of freedom. There are additional shortcomings of the prevalent data analytical strategy, such as violated model assumptions (e.g., normally distributed and homoscedastic residuals; Gelman and Hill 2006). In sum, these statistical issues discussed presumably lead to inconsistent results, which have been reported in the literature.

It is important to note that there seems to be a confirmation bias in psychology. This bias usually occurs if subjects are asked to evaluate ambiguous evidence and see their initial expectations confirmed. Equipped with the hypothesis that the relation between intelligence and creativity is weaker above some thresholds, and given the inconclusive literature with partial support for a threshold, researchers are more likely to find that a threshold exists rather than contemplating why their results are at odds with what seems to be a compelling and positive result. Indeed, it is likely that a critical reader suspects those studies that are unable to find a threshold were somewhat flawed, maybe suffering from methodological deficiencies such as small sample sizes, inadequate measures, or other biases. This suspicion is very likely justified since most of the research—including the “positive” findings—suffers from these shortcomings (Ioannidis 2005). In the same vein, researchers who find themselves confronted with a negative result might feel the urge to try searching a little harder to escape these allegations, or to get their results published more easily (Bakker et al. 2012). We are afraid this explanatory bias helps the threshold hypothesis to escape extinction. 

### 9.3. How Should We Approach the Threshold Hypothesis?

We wanted to shed light on a research question that has led to diverging results for over 50 years. We applied analytical strategies that have been used previously—such as the test of heteroscedasticity and the segmented regression analysis—but both approaches usually rely on manifest variables. Therefore, we proposed local structural equation modeling as an additional novel and powerful analytical tool for a continuous treatment of moderators. However, in LSEM, large sample sizes are required to estimate models at each focal point. In the case of the intelligence-creativity threshold hypothesis, this implies that large sample sizes (about *N* = 150; e.g., Muthén and Muthén 2002) are needed at the tails of distribution, which further increases sampling difficulties for normally distributed variables, such as creativity and intelligence. In contrast to other methods, LSEM allows for the detection of non-linear trends and an investigation into the origins of violations of measurement invariance (e.g., Hartung et al. 2020; Olaru et al. 2019).

With the present manuscript, we sought to demonstrate that the search for a specific threshold between intelligence and creativity is a wild goose chase. With that said, we do not want to discourage theoretically well-informed studies that are conducted with the necessary methodological rigor. However, we remain skeptical that a profound theoretical basis exists for further assuming a threshold or a non-linear relationship. In sum, there is no convincing evidence—theoretically or analytically—for the existence of a threshold in the relation between creativity and intelligence. Intelligence is definitely relevant for producing divergent ideas, but its relation appears linear across the continuum of intelligence. If measured broadly, the magnitude of the correlation also seems to fall within an expectable range, which mitigates prior concerns on the strength of the relation between intelligence and creativity. We assume that differentiation will not appear for other factors of creativity (e.g., originality) and intelligence (e.g., crystallized intelligence; Sligh et al. 2005). Nevertheless, studying such aspects in the future—for example, the relation between general retrieval ability, creative retrieval, and crystallized intelligence (e.g., Forthmann et al. 2019), or the overlap between fluency and originality—is interesting to further our understanding of cognitive abilities and its relationship with creativity. 

## Figures and Tables

**Table 1:** Previous investigations in the relation of creativity and intelligence.

Study	Sample	Analytical Method	Measures of Creative Ability (DT)	Measures of Intelligence	Results	Threshold (*z*-Standardized)
Guilford and Christensen (1973)	360 (students)	Scatterplots	10 verbal and figural DT tests ^1^	e.g., Stanford Achievement Test	No Threshold	-
Fuchs-Beauchamp et al. (1993)	496 (pre-schoolers)	Correlations in two IQ groups	Thinking Creatively in Action and Movement ^2^	e.g., Stanford-Binet Intelligence Scale ^8^	Threshold	1.33
Sligh et al. (2005)	88 (college students)	Correlations in two IQ groups	Finke Creative Invention Task ^3^	KAIT ^9^	No Threshold	-
Preckel et al. (2006)	1328 (students)	Correlations and Multigroup CFA	BIS-HB ^4^	BIS-HB ^4^	No Threshold	-
Holling and Kuhn (2008)	1070 (students)	Multigroup CFA	BIS-HB ^4^	Culture Fair Test ^10^	No Threshold	-
Cho et al. (2010)	352 (young adults)	Correlations in two IQ groups	Torrance Test ^5^	e.g., WAIS ^11^	Threshold	1.33
Jauk et al. (2013)	297 (adults)	SRA	Alternate Uses and Instances ^6^	Intelligence-Structure-Battery ^12^	Threshold	−1.00 to 1.33
(Karwowski and Gralewski (2013)	921 (students)	Regression analysis and CFA	Test for Creative Thinking-Drawing Production ^7^	Raven’s Progressive Matrices ^13^	Threshold	1.00 to 1.33
Shi et al. (2017)	568 (students)	among others SRA	Torrance Test ^5^	Raven’s Progressive Matrices ^13^	Threshold	0.61 to 1.12

SRA = Segmented Regression Analysis; CFA = Confirmatory Factor Analysis, ^1^ Wilson et al. (1954); ^2^ Torrance (1981); ^3^ Finke (1990); ^4^ BIS-HB = Berlin Intelligence Structure Test, Jäger et al. (1997); ^5^ Torrance (1999); ^6^ Jauk et al. (2013); ^7^ Urban (2005); ^8^ Terman and Merill (1973); ^9^ KAIT = Kaufman Adolescent and Adult Intelligence Test, Kaufman and Kaufman (1993); ^10^ Cattell and Cattell (1960); ^11^ WAIS = Wechsler Adult Intelligence Scale, Wechsler (1981); ^12^ Arendasy et al. (2004); ^13^ Raven et al. (2003).

## Footnotes

1 We understand creativity as the ability to produce divergent ideas; thus, we do not further distinguish between creativity and divergent thinking for the purpose of this paper and use the terms interchangeably from now on.

## Supplementary Materials

The following are available online at https://www.mdpi.com/2079-3200/8/4/38/s1, Table S1: Descriptive Statistics for all Indicators, Figure S1: Correlations and Bivariate Scatterplots between Manifest Scores, Figure S2: Scatterplot and Heteroscedasticity Plot in Study 1: Originality, Figure S3: Segmented Regression Analysis in Study 1: Originality, Figure S4: Factor Variances at each Focal Point along the Intelligence Continuum in Study 1: Originality, Figure S5: Loadings at the Focal Points in Dataset 1, Figure S6: Loadings at the Focal Points in Dataset 2.
