# Supplementary material for: A Reappraisal of the Threshold Hypothesis of Creativity and Intelligence
Source: J Intell. 2020 Nov 11;8(4):38. doi: 10.3390/jintelligence8040038 (PMC7709632; doi:10.3390/jintelligence8040038)
Supplement: Supplementary file 1 [file jintelligence-08-00038-s001.pdf]

**A Reappraisal of the Threshold Hypothesis of Creativity and Intelligence**

**Supplementary Material**

**Table S1**

*Descriptive Statistics for all Indicators*

| Dataset | Construct | Task | *N* | *Mean* (*SD*) |
|---|---|---|---|---|
| 1 | DT | Shoe | 453 | 6.79 (3.26) |
| | | Bottle | 453 | 7.00 (2.87) |
| | | Umbrella | 453 | 6.71 (2.78) |
| | IQ | Numerical | 451 | 9.39 (4.55) |
| | | Verbal | 454 | 14.72 (4.28) |
| | | Retrieval | 455 | 8.30 (3.76) |
| 2 | DT | SA | 421 | 7.24 (2.07) |
| | | NI | 425 | 1.97 (0.67) |
| | | IN | 423 | 0.80 (0.36) |
| | | FF | 427 | 5.64 (1.59) |
| | | CO | 424 | 1.79 (0.35) |
| | | RF | 415 | 8.51 (2.16) |
| | IQ | Verbal and Figural | 423 | 0.62 (0.15) |

*Note.* Dataset 2: Fluency indicators are SA (similar attributes), IN (inventing names), FF (figural fluency), and RF (retrieval fluency). CO (combining objects) and NI (nicknames) are indicators of Originality that were only instructed for originality. Fluency indicators were rated regarding the quantity of correct responses, originality was rated regarding the quality of a single given response.

**Figure S1**

*Correlations and Bivariate Scatterplots Between Manifest Scores*

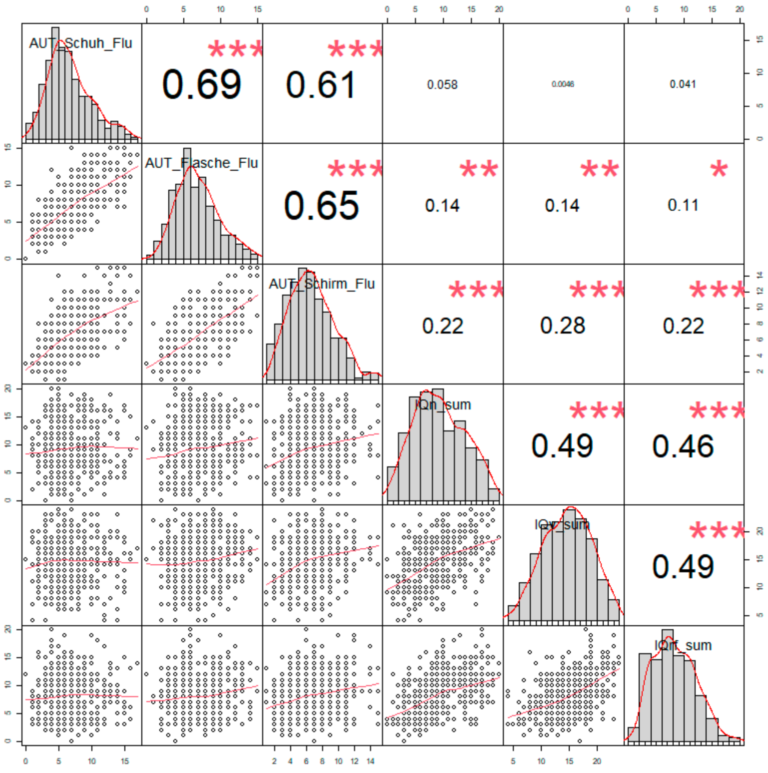


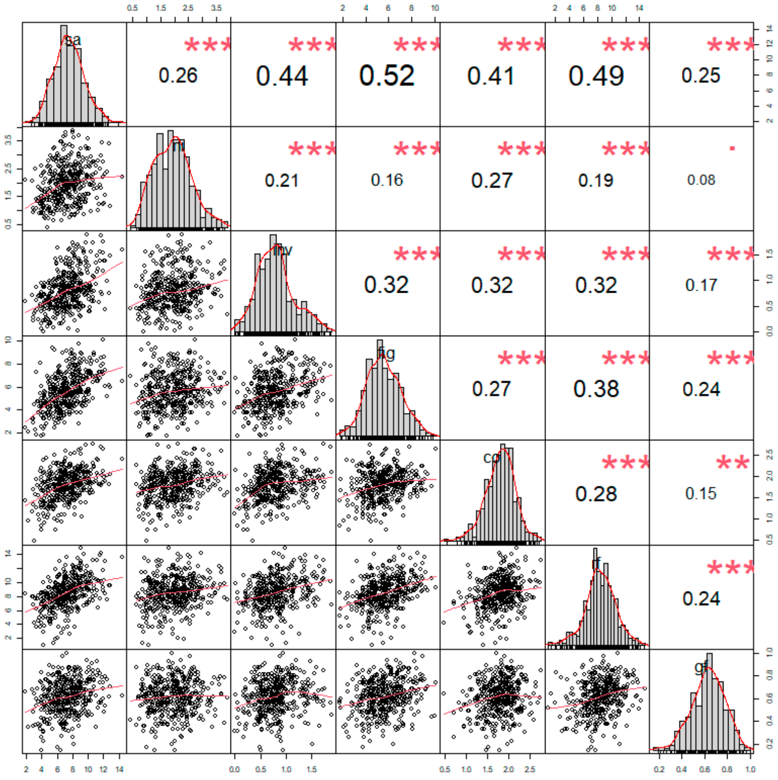


*Note.* Datasets 1 upper figure and 2 lower figure; * < .05; ** < .01; *** < .001.

**Figure S2**

*Scatterplot and Heteroscedasticity Plot in Study 1: Originality.*

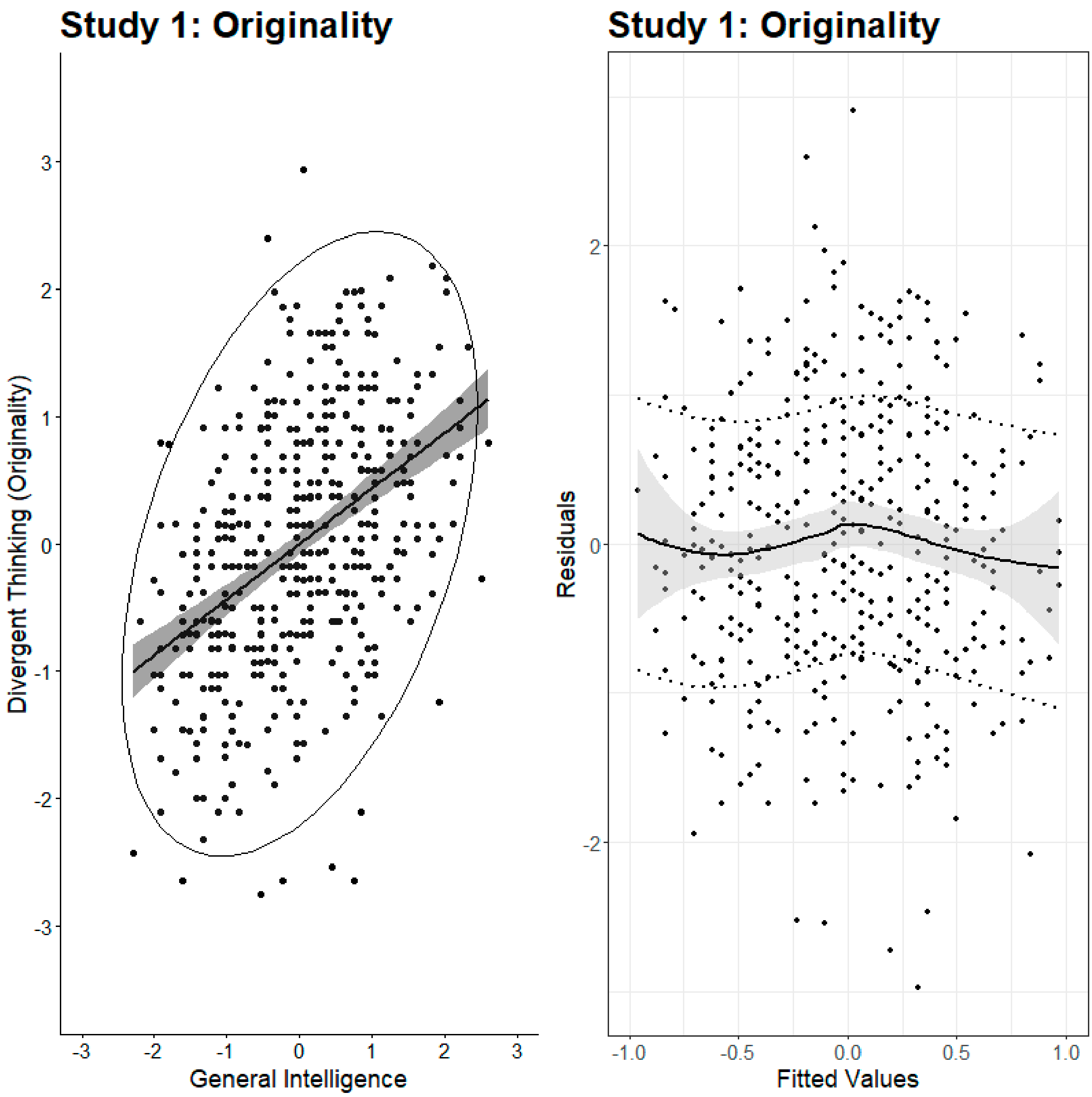


*Note.* Scatterplot (including a 0.95 confidence interval and an ellipse) for the correlation between divergent thinking and intelligence are presented upper plots. Heteroscedasticity plots (lower plots) include standard errors (grey) and standard deviations of the fitted values (dashed line).

**Figure S3**

*Segmented Regression Analysis in Study 1: Originality.*

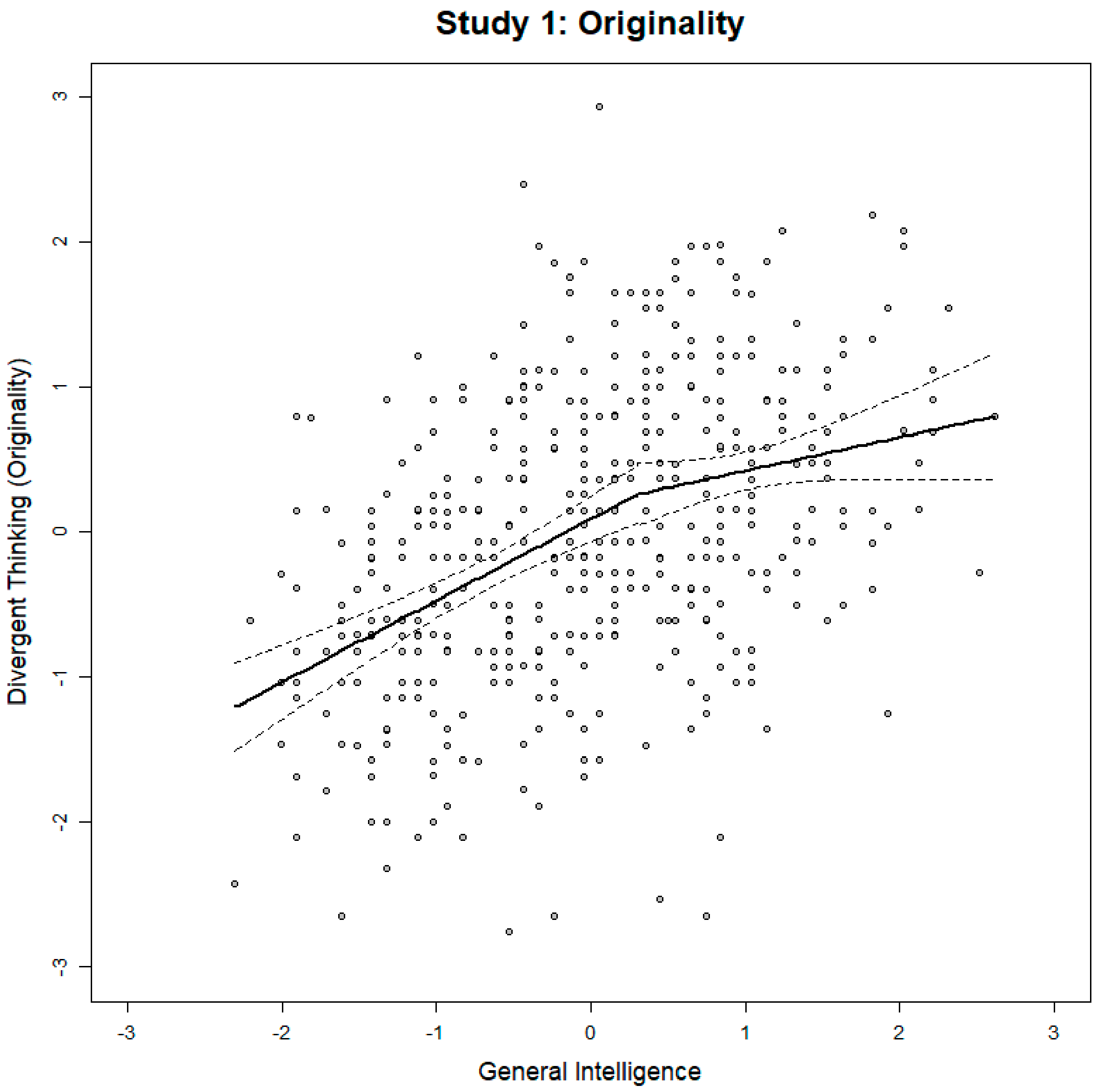


*Note.* Breakpoint for the relation between general intelligence and divergent thinking. Dotted line = 95% CI.

**Figure S4**

*Factor Variances at each Focal Point along the Intelligence Continuum in Study 1: Originality.*

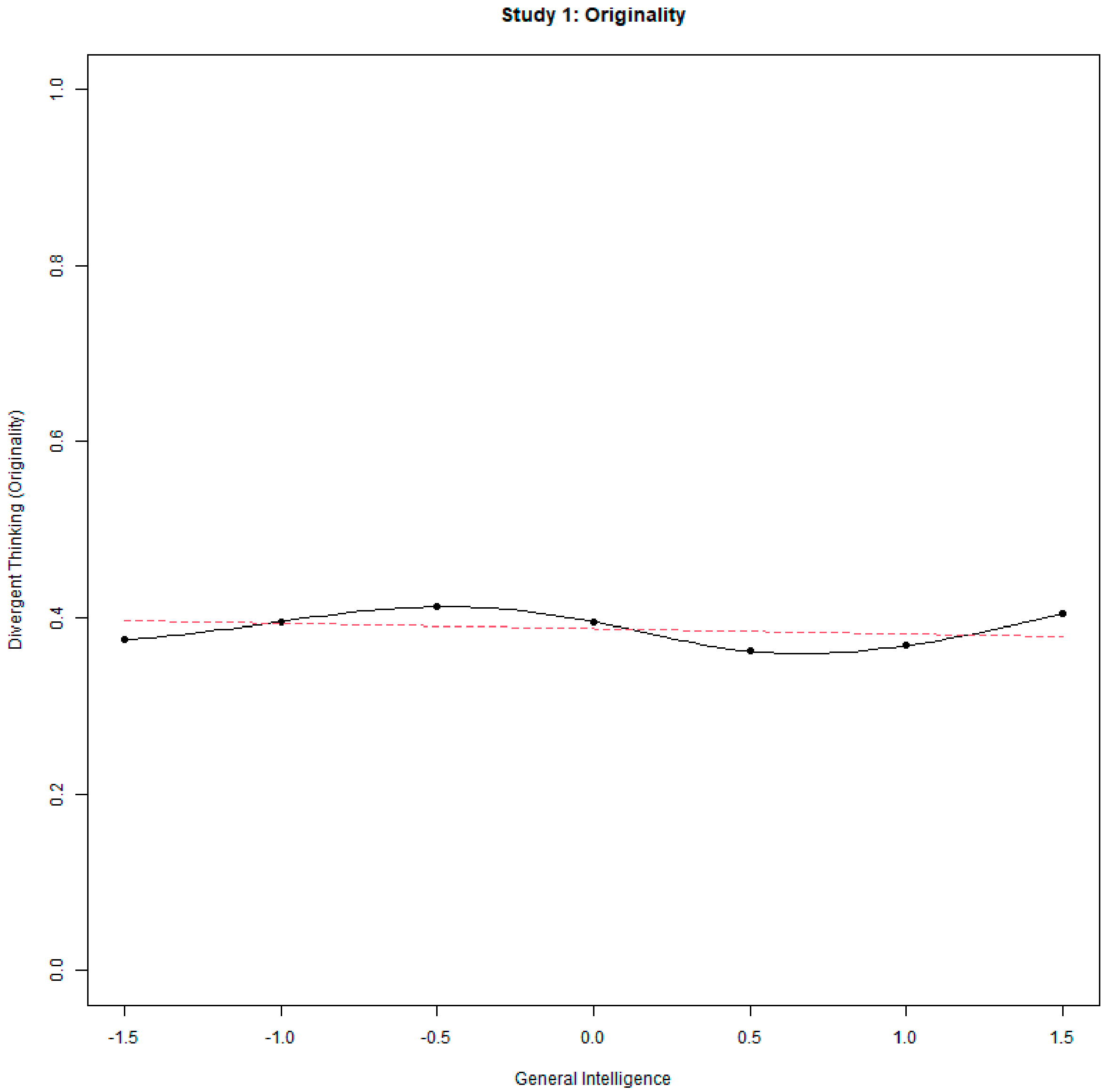

**Figure S5**

*Loadings at the Focal Points in Dataset 1.*

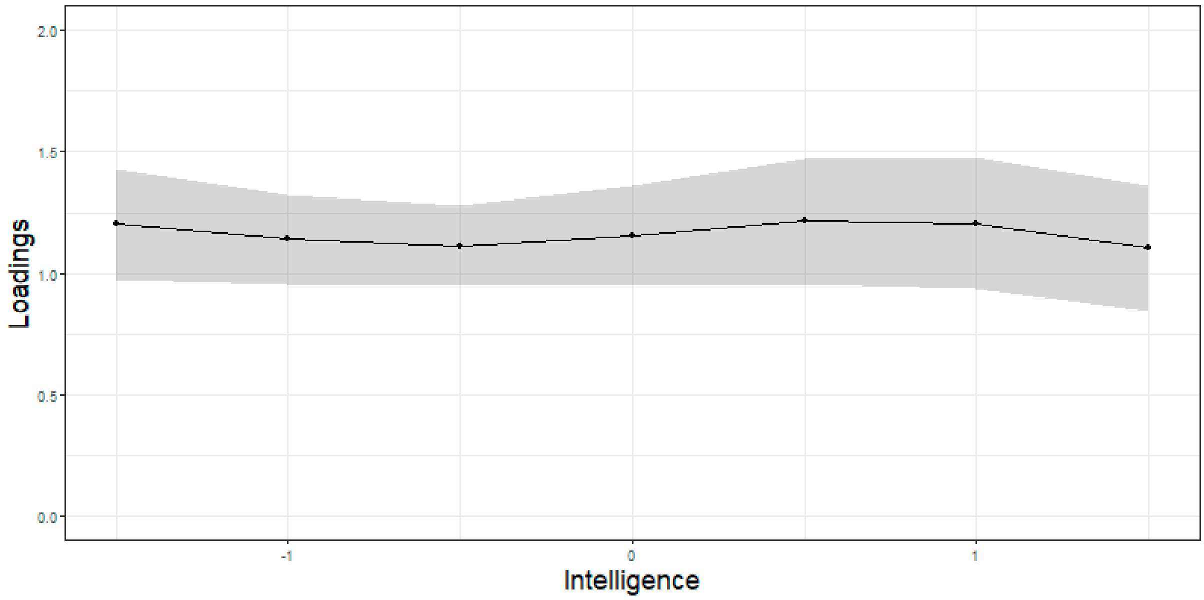


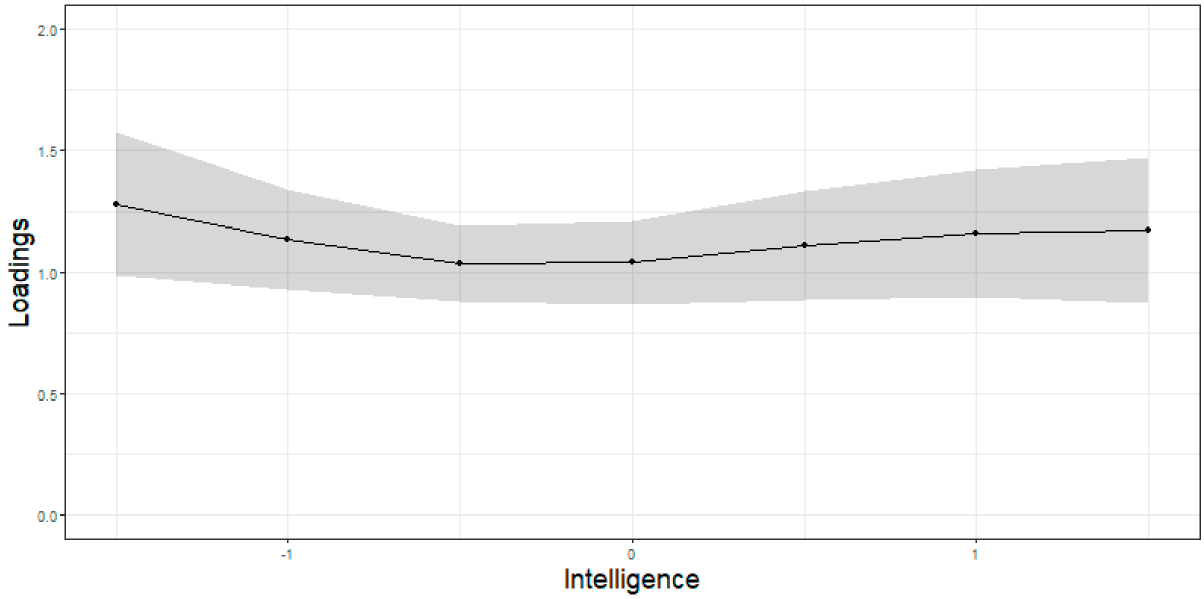


*Note.* Item 2 displayed in the upper figure and item 3 in the lower figure.

**Figure S6**

*Loadings at the Focal Points in Dataset 2.*

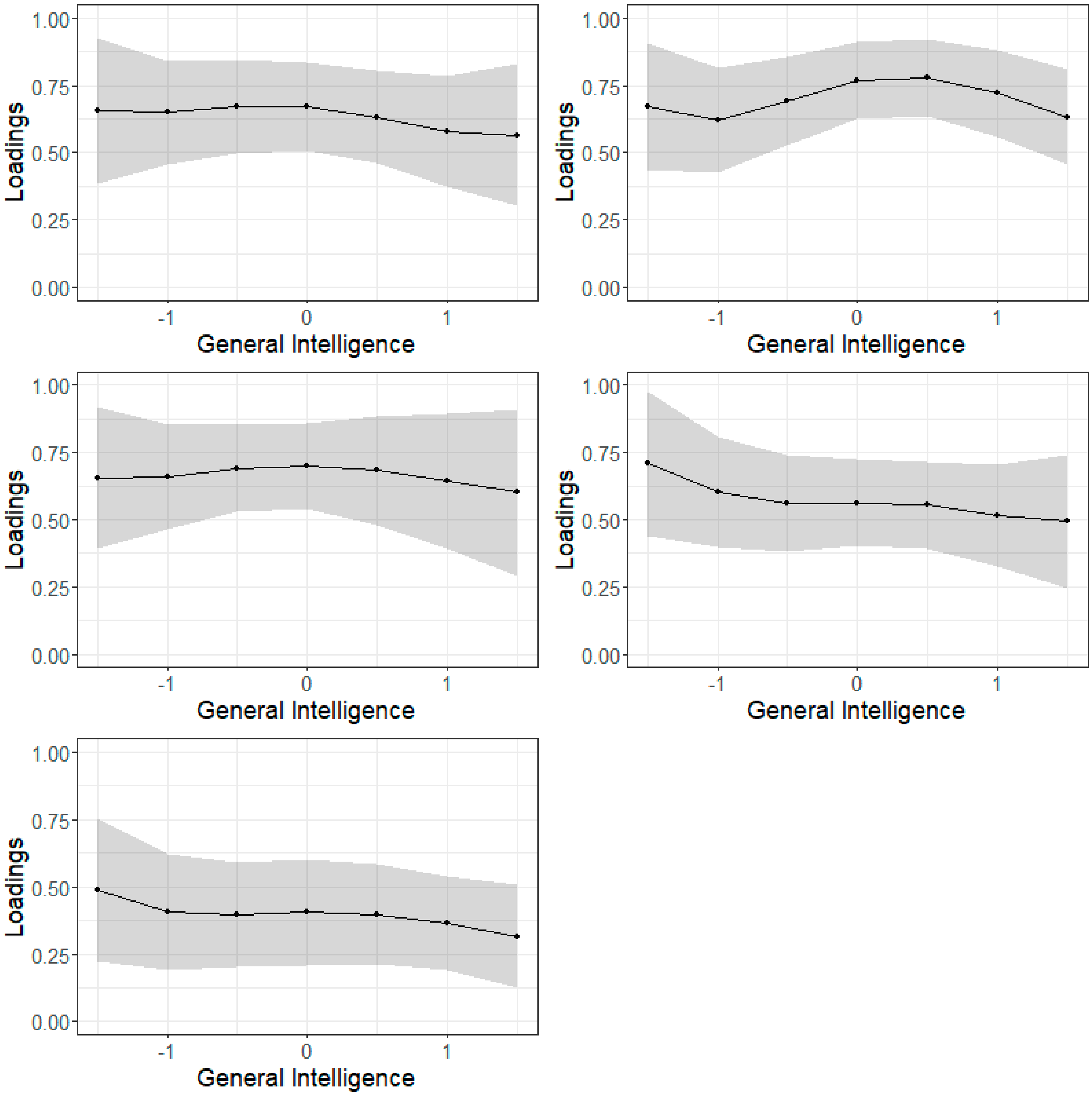


*Note.* From upper left: item 2, 3, 4, 5 and 6
